# Contrast-Enhanced, Fluoroscopically Guided Percutaneous Endoscopic Gastrostomy Tube Placement for the High-Risk Patient

**DOI:** 10.14309/crj.0000000000000740

**Published:** 2022-01-17

**Authors:** Preeti Prakash, Andrew Su, Leona Mason, James H. Tabibian

**Affiliations:** 1 Department of Internal Medicine, Ronald Reagan UCLA Medical Center, Los Angeles, CA; 2 Vatche and Tamar Manoukian Division of Digestive Diseases, David Geffen School of Medicine at UCLA, Los Angeles, CA; 3 Division of Infectious Diseases, Department of Medicine, Olive View-UCLA Medical Center, Sylmar, CA; 4 Division of Gastroenterology, Department of Medicine, Olive View-UCLA Medical Center, Sylmar, CA

## Abstract

Percutaneous endoscopic gastrostomy (PEG) tubes can facilitate enteric feeding in patients with severe malnutrition but may be technically challenging to place. We present a man with disseminated tuberculosis and severe cachexia refractory to oral intake and nasogastric tube placement. PEG placement was initially deemed high-risk, through endoscopic, interventional radiologic, or surgical approach, because of severe cachexia and dilated bowel loops interposed between the abdominal wall and stomach. We describe a novel, minimally invasive technique to enhance safety and feasibility of PEG placement, which led to significant improvement in nutritional status and facilitated successful response to tuberculosis therapy.

## INTRODUCTION

Percutaneous endoscopic gastrostomy (PEG) tubes can play a vital role in various clinical scenarios, such as in severely malnourished patients refractory to or unable to tolerate oral intake or temporary forms of enteric nutrition, eg, nasogastric tube feeding. Severe malnutrition is seen frequently in advanced tuberculosis (TB) and can lead to poor response and increased mortality while on intensive treatment.^1,2^ Correction of underlying malnutrition with PEG feeding can significantly improve outcomes; however, PEG tube placement can be technically difficult or infeasible in some cases.

## CASE REPORT

A 70-year-old homeless man was hospitalized for disseminated isoniazid-resistant TB and severe cachexia. He weighed 56 kg (body mass index of 23) on admission, which declined to 44 kg (body mass index of 18.2) over the next month despite appropriate therapy. Serum albumin and prealbumin were 1.5 g/dL and 8 mg/dL, respectively. He could not meet his daily caloric needs orally or with nutritional supplementation despite attempts at optimization. A nasogastric tube was trialed, but he did not tolerate extended placement and refused repeat placement.

**Figure 1.**
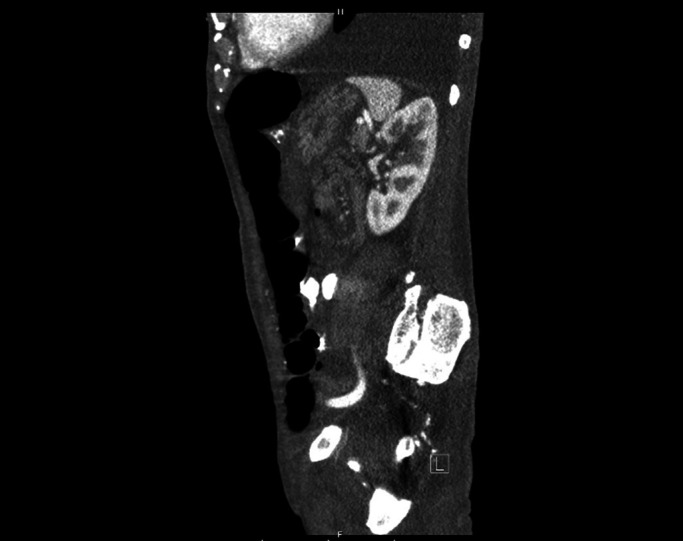
Abdominal computed tomography (sagittal projection) showing dilated loops of bowel and posterior displacement of a decompressed/retracted stomach.

It was the consensus of the primary TB team, the nutrition service, and the patient that a PEG tube would be his best option. However, 2 separate abdominal computed tomography (CT) scans showed diffusely dilated loops of the colon and, to a lesser extent small bowel, interposed between the abdominal wall and the stomach. In addition, much of the stomach seemed subcostal, possibly secondary to cephalad displacement from the distended bowels (Figure 1). Bowel distention did not improve despite correction of electrolyte abnormalities, aggressive bowel regimen, and 4-L polyethylene glycol purgation. Gastroenterology was consulted for PEG. On review of imaging, the window for PEG placement was considered unsuitable, with high risk of bowel puncture. Interventional radiology was consulted but similarly felt that there was no viable percutaneous window. General surgery determined the patient to not be a surgical gastrostomy tube (G-tube) candidate because of increased perioperative risk.

**Figure 2.**
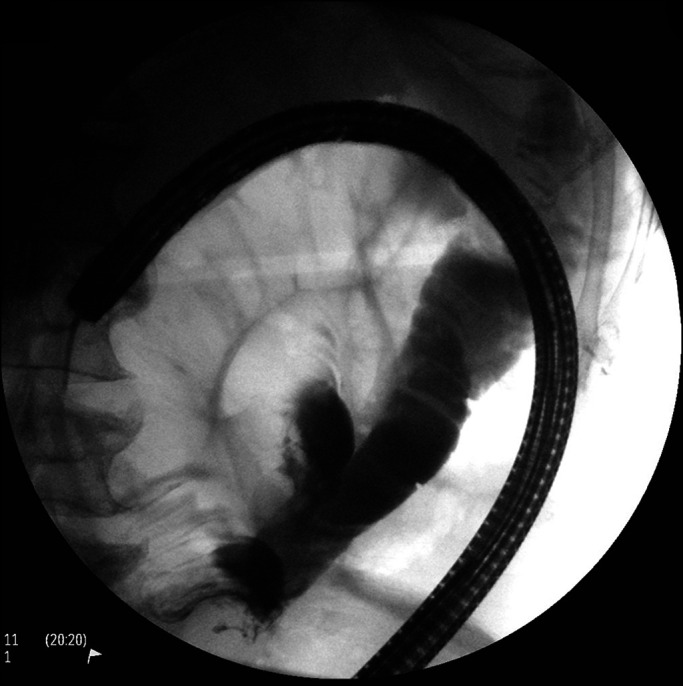
Colonoscopic preparation prepercutaneous endoscopic gastrostomy tube placement.

**Figure 3.**
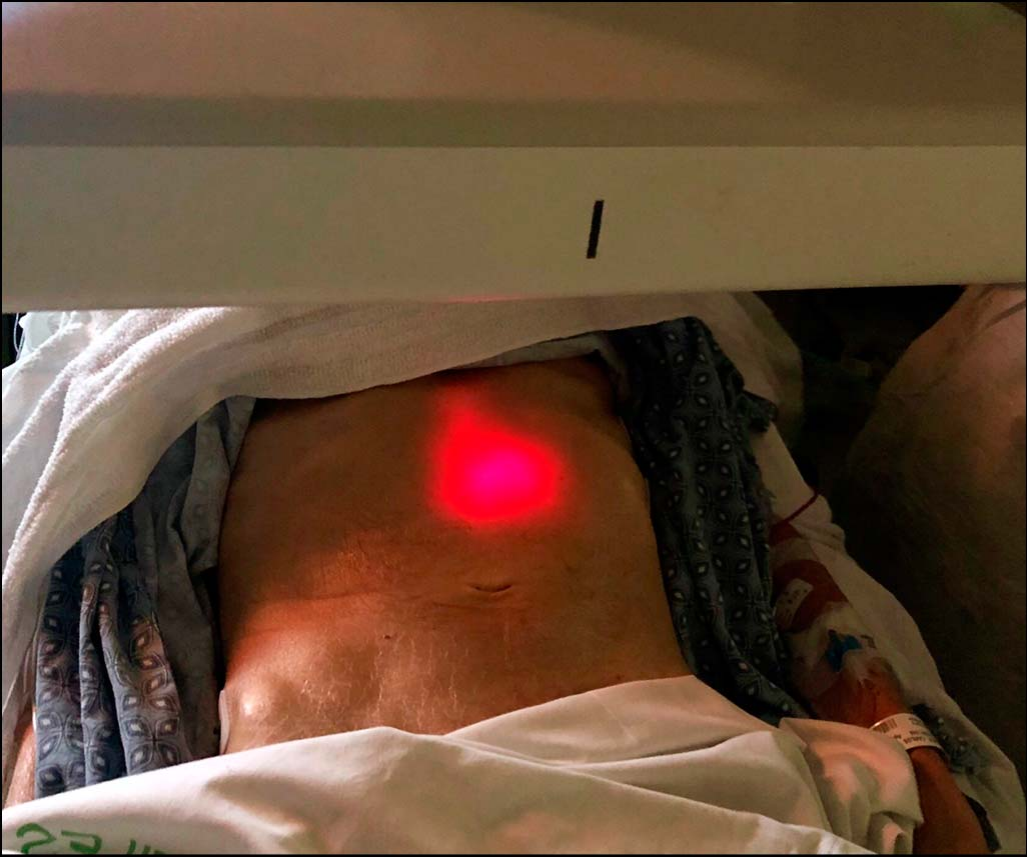
With the stomach insufflated endoscopically, transillumination was confirmed to the left of midline and caudal to the inferior costal margin, in conventional anatomic location.

**Figure 4.**
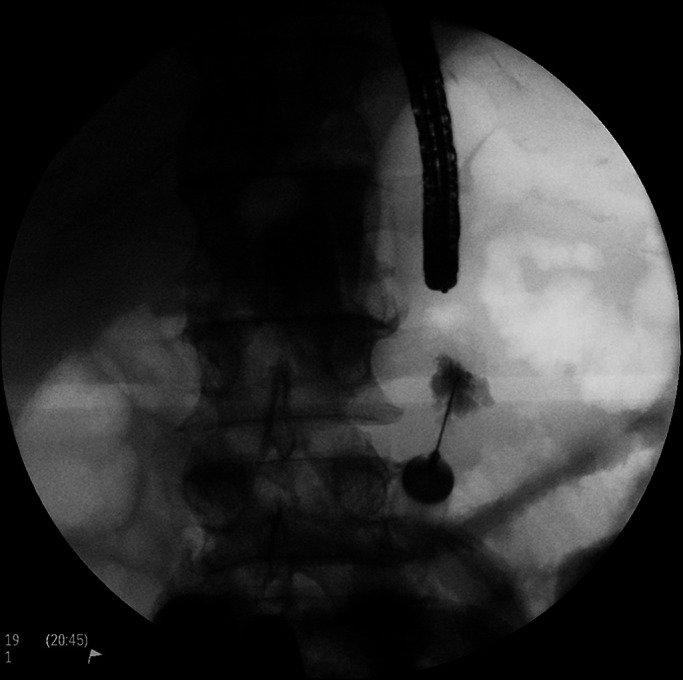
Confirmation of percutaneous endoscopic gastrostomy tube placement site.

**Figure 5.**
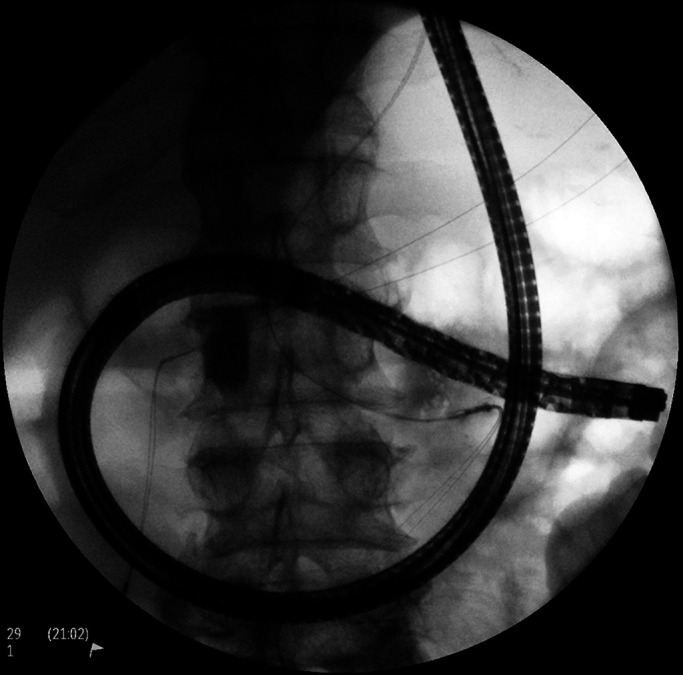
Anterograde enteroscopy performed to further confirm the absence of small bowel puncture before placement of the percutaneous endoscopic gastrostomy tube by conventional pull technique.

After an extensive multidisciplinary discussion, it was proposed that the patient undergo contrast-enhanced, fluoroscopically guided PEG placement. After acquisition of a scout fluoroscopic image, using water and minimal CO_2_ insufflation, an adult colonoscope was advanced through the distended colon to the cecum. During withdrawal, decompression was performed and iohexol injected throughout the colon, beginning in the cecum, to provide fluoroscopic opacification (Figure 2). Esophagogastroduodenoscopy was performed next, with normal findings. Repeat fluoroscopy revealed a relatively decompressed colon containing abundant contrast, as intended; small bowel distention seemed slightly improved. The stomach was insufflated, and transillumination was confirmed to the left of midline, caudal to the inferior costal margin, per conventional technique (Figure 3). A 20-gauge needle was advanced percutaneously at this site with simultaneous injection of a solution of 1% lidocaine mixed 50:50 with sterile iohexol. There was no bowel opacification on fluoroscopy, and the needle tip was seen entering the stomach endoscopically. The catheter needle was thus introduced along this tract into the stomach (Figure 4). After a guidewire was passed and endoscopically grasped, another syringe of the aforementioned solution was attached to the catheter needle end and injected as the needle was pulled out. Again, no bowel opacification was seen. Given the dilated loops of the small bowel, enteroscopy using an ultraslim (PCF-PH190L/I) colonoscope was performed as an added measure to rule out the possibility of intestinal puncture (Figure 5). This was unremarkable, and as positioning seemed appropriate, a 20Fr tube was ultimately placed by pull technique.

**Figure 6.**
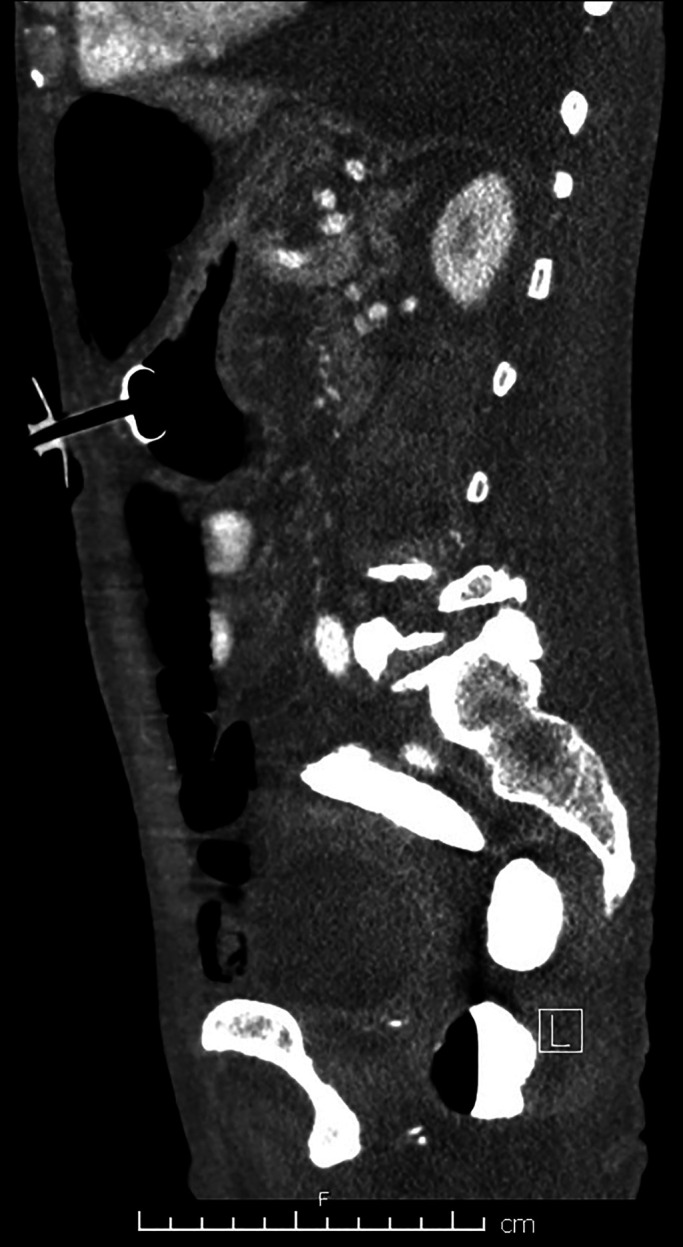
Postprocedure abdominal computed tomography (sagittal projection) confirming successful placement of the percutaneous endoscopic gastrostomy tube.

Abdominal CT was repeated before initiating feeds and confirmed successful PEG placement without bowel traversal (Figure 6). The patient underwent 8 weeks of intensive TB treatment, after which excellent response was observed, weight improved to 51 kg, albumin to 2.7 g/dL, and prealbumin to 30 mg/dL.

## DISCUSSION

Malnutrition is an indication for G-tube placement in patients with TB.^3^ G-tubes can be placed endoscopically, radiologically, or surgically.^4–7^ Regarding the endoscopic approach (PEG), there are standard steps to minimize morbidity.^8^ These include endoscopic transillumination, one-to-one finger indentation, and the “safe tract” syringe aspiration technique during needle insertion.^9^ In addition, contraindications to and factors that may interfere with safe PEG placement should be recognized. These include difficulty with transillumination (eg, morbid obesity, large-volume ascites, and peritoneal carcinomatosis), inability to pass an endoscope (eg, obstructing oropharyngeal cancer), and systemic issues (eg, unaddressed coagulopathy).

Bowel distention can pose a significant challenge vis-à-vis PEG placement by any approach. Internal organ injury, particularly small bowel or colon, has been described^10,11^ with resultant peritonitis.^12,13^ Laparoscopic-assisted PEG is a hybrid option for patients with severely dilated loops of bowel with interposition between the stomach and abdominal wall^14^; in this case, the patient was deemed too high risk even for this approach. Alternative techniques have been tried to facilitate PEG placement, including assistance with transabdominal ultrasound, CT, and endoscopic ultrasound.^15–20^ We have described a novel technique that allows for minimally invasive placement of a PEG tube in a high-risk patient and in this case played a critical role in TB treatment success and overall clinical recovery.

## DISCLOSURES

Author contributions: P. Prakash drafted and revised the manuscript. A. Su drafted the manuscript and acquired images. L. Mason edited and revised the manuscript. JH Tabibian acquired images, edited and revised the manuscript, and is the article guarantor.

Financial disclosure: None to report.

Informed consent was obtained for this case report.
